# Detecting Causality in Multivariate Time Series via Non-Uniform Embedding

**DOI:** 10.3390/e21121233

**Published:** 2019-12-16

**Authors:** Ziyu Jia, Youfang Lin, Zehui Jiao, Yan Ma, Jing Wang

**Affiliations:** 1 School of Computer and Information Technology, Beijing Jiaotong University, Beijing 100044, China; ziyujia@bjtu.edu.cn (Z.J.); yflin@bjtu.edu.cn (Y.L.); zhjiao@bjtu.edu.cn (Z.J.); 2 Division of Interdisciplinary Medicine and Biotechnology, Department of Medicine, Beth Israel Deaconess Medical Center/Harvard Medical School, Boston, MA 02215, USA; dr.yan.ma@gmail.com

**Keywords:** causal analysis, non-uniform embedding, multivariate time series, conditional mutual information

## Abstract

Causal analysis based on non-uniform embedding schemes is an important way to detect the underlying interactions between dynamic systems. However, there are still some obstacles to estimating high-dimensional conditional mutual information and forming optimal mixed embedding vector in traditional non-uniform embedding schemes. In this study, we present a new non-uniform embedding method framed in information theory to detect causality for multivariate time series, named LM-PMIME, which integrates the low-dimensional approximation of conditional mutual information and the mixed search strategy for the construction of the mixed embedding vector. We apply the proposed method to simulations of linear stochastic, nonlinear stochastic, and chaotic systems, demonstrating its superiority over partial conditional mutual information from mixed embedding (PMIME) method. Moreover, the proposed method works well for multivariate time series with weak coupling strengths, especially for chaotic systems. In the actual application, we show its applicability to epilepsy multichannel electrocorticographic recordings.

## 1. Introduction

In recent years, various time series analysis methods have been proposed to detect interactions between complex systems [1,2,3]. The study of causality, in particular, has attracted wide attention of researchers. There are two classic methods in the time series causal analysis: Granger causality [4] and transfer entropy [5,6]. Both methods are based on time series prediction for causal analysis. In addition, the relationship between Granger causality and transfer entropy is demonstrated [7]: the two methods are equivalent under Gaussian assumptions. Furthermore, Hlavackova–Schindler [8] extends the equivalence of the two causality methods for generalized Gassian processes which satisfy some additional conditions on probability density distributions.

With the development of multivariate state space reconstruction, different embedding schemes [9,10,11,12,13] are used in Granger causality and transfer entropy. The common idea of those embedding schemes is to reconstruct the past of the whole system represented by all variables with reference to the target variable, in order to form a mixed embedding vector containing the most significant past variables to explain the target variable. Non-uniform embedding schemes are proposed to solve the problems of redundancy and arbitrariness in uniform embedding schemes [11,14]. Vlachos et al. propose a causality measure for bivariate variables based on the mixed embedding scheme: the conditional mutual information from mixed embedding (MIME) [15]. Kugiumtzis et al. extend the MIME method to multivariable time series and form the partial MIME (PMIME) method [16]. The PMIME method successfully solves the problem of detecting direct causality in multivariable time series. In addition, it is gradually applied to complex systems such as physiology [17,18] and finance [19,20].

Although the causal analysis using non-uniform embedding has practical advantages, there are still some key shortcomings that need to be overcome. One shortcoming is the curse of dimensionality, which makes the estimation of mutual information inaccurate as the dimension of the embedded space increases [21,22,23,24]. Another shortcoming is related to the mixed embedding vector. The greedy strategy uses a sequential forward approach to select the lagged variables and finally form the mixed embedding vector [11,15,16]. That is to say, the lagged variables that have been embedded will not be changed in the mixed embedding vector. As the iteration increases, more lagged variables are selected until the final mixed embedding vector is formed. Therefore, the inaccuracy of the initial embedding will have a large impact on the results. The above shortcomings will be highlighted when there are multivariate time series of weak causal coupling strengths in practical applications.

In order to overcome the above shortcomings, we propose a new non-uniform embedding method named LM-PMIME for multivariate time series based on the low-dimensional approximation of conditional mutual information(CMI) and the mixed search strategy. The main contribution of the proposed method is to reduce the dimension of the embedded space by replacing the original estimate with a low-dimensional approximation of CMI. In addition, a mixed strategy, which has taken the place of the greedy strategy, was adopted as an embedded strategy to optimize the initial embedding. The proposed method works well for multivariate time series with weak coupling strengths.

The rest of the paper is organized as follows. In Section 2, we propose the multivariate non-uniform embedding in accordance with the low dimensional approximation of CMI and a mixed search strategy. In Section 3, we perform the simulation experiments in order to verify the effectiveness of the LM-PMIME method. In Section 4, by analyzing the electrocorticographic (ECoG) recordings from an epileptic patient, the applicability of the proposed method to actual data is shown. Finally, a summary is presented in Section 5.

## 2. Method

In this section, we first introduce the traditional PMIME method. Then we expound a low dimensional approximation of CMI and a mixed search strategy. Finally, we present the LM-PMIME method for multivariable non-uniform embedding.

### 2.1. PMIME Method

The PMIME method, a generalization of the MIME method for bivariate variables [15], is developed by Kugiumtzis et al. [16] to detect the directional coupling in multivariable time series. Let *K* variables $X,Y,Z_{1},\ldots ,Z_{K-2}$ constitute an overall dynamical system $\left\{x_{t},y_{t},z_{1,t},\ldots ,z_{K-2,t}\right\}{}_{t=1}^{n}$. Suppose that the driving subsystem is *X* and the target subsystem is *Y*. In other words, the current value of variable *Y* is affected by the past of variable *X*. $Z=\{Z_{1},\ldots ,Z_{K-2}\}$ represent the remaining subsystems.

We estimate the causal effect of *X* on *Y* conditioned by $Z=\{Z_{1},\ldots ,Z_{K-2}\}$. It is necessary to form a set of variables representing the past of the subsystems. The lags of *X*, *Y* and *Z* are sought within a range given by a maximum lag for each variable, e.g., $L_{x}$ for *X* and $L_{y}$ for *Y*. $W_{t}$ is defined as the set of all lagged variables at time *t*, containing the parts $x_{t},x_{t-1},\ldots ,x_{t-L_{x}}$ of *X* and the same for *Y* and *Z*. It is usually assumed that the maximum lag *L* for all variables is the same ($L_{x}=L_{y}=L_{z}$). The larger the value of *L*, the more lagged variables are included in $W_{t}$. The key step of the PMIME method is to form the mixed embedding vector $v_{t}\in W_{t}$ using non-uniform embedding. The stopping criterion and greedy selection are applied to the process of embedding. The detailed method is described below as follows:

1. An empty embedding vector $v{}_{t}^{0}=\emptyset$ is initialized.
2. At the first iteration *k* = 1, the embedding vector $w{}_{t}^{1}\in W_{t}$ is selected most related to $y_{t}$:
  (1) $$w{}_{t}^{1}=\underset{w\in W_{t}}{\arg\max}I\left(y_{t};w\right),$$
  where I(.) represents mutual information. Mutual information is estimated by the k-nearest neighbors (k-NNs) method. Then we have $v{}_{t}^{1}=\left[w{}_{t}^{1}\right]$. At the same time, $w{}_{t}^{1}$ is removed from $W_{t}$.
3. At the iteration k>1, the mixed embedding vector is augmented by the component $w{}_{t}^{k}$ of $W_{t}$, giving most information about $y_{t}$ additionally to the information already contained in $v{}_{t}^{k-1}=\left[w{}_{t}^{1},\ldots ,w{}_{t}^{k-1}\right]$. $w{}_{t}^{k}$ will be selected by a standard through calculating the maximum value of the conditional mutual information, $w{}_{t}^{k}={\arg\max}_{w\in W_{t}}I\left(y_{t};w|v{}_{t}^{k-1}\right)$, i.e., at the iteration k=2, $w{}_{t}^{2}={\arg\max}_{w\in W_{t}}I\left(y_{t};w|v{}_{t}^{1}\right)$, where the CMI is calculated by the k-NNs estimator, and the mixed embedding vector is $v{}_{t}^{2}=\left[w{}_{t}^{1},w{}_{t}^{2}\right]$. By using greedy forward method, each $w{}_{t}^{k}$ will be embedded in the already embedded vector $v{}_{t}^{k-1}$ until the process stops. The termination criterion is quantified as:
  (2) $$I\left(y_{t};v{}_{t}^{k-1}\right)/I\left(y_{t};v{}_{t}^{k}\right)>A,$$
  where the threshold A<1 and the general value of *A* is 0.95 or 0.97 in [15,16]. That is, the additional information of $w{}_{t}^{k}$ selected at the iteration *k* is not large enough. The embedding process will stop and we have the mixed embedding vector $v_{t}=v{}_{t}^{k-1}$. Any combination of the lagged variables $X,Y,Z_{1},\ldots ,Z_{K-2}$ may be included in $v_{t}$.
4. To calculate the causality strength of *X* on *Y* conditioned by the other variables in *Z*, the index is defined as
  (3) $$R{}_{X\to Y|Z}=\frac{I(y_{t};v{}_{t}^{x}|v{}_{t}^{y},v{}_{t}^{z})}{I(y_{t};v{}_{t})},$$
  where $v{}_{t}^{x}$ represents the component of *X* in $v{}_{t}$. It is the same with $v{}_{t}^{y}$ and $v{}_{t}^{z}$. The causal effect of *X* to *Y* depends on the components of *X* in $v{}_{t}^{x}$.

### 2.2. The Proposed Method

#### 2.2.1. Low Dimensional Approximation of CMI

As the dimension of the mixed embedding vector increases, the estimation of CMI becomes less reliable. Because of an increasing volume of state space, the estimation of entropy rates progressively decreases towards zero [15]. Therefore, in order to overcome the problems caused by computing high-dimensional CMI, the low-dimensional approximation of CMI is a better alternative. The low-dimensional approximation can improve the accuracy of conditional mutual information estimation and reduce the computational cost.

The low-dimensional approximation of CMI is studied by researchers in the feature selection [25,26,27,28,29,30,31]. Brown et al. [23] emphasize that lots of feature selection heuristics are all approximate iterative maximizers of the conditional likelihood. Consequently, the methods are summarized as a parameterized general standard:

(4) $$I\left(w;y_{t}\right)-\beta \sum_{w_{i}\in v_{t}}I\left(w;w_{i}\right)+\gamma \sum_{w_{i}\in v_{t}}I\left(w;w_{i}|y_{t}\right),$$

where the difference between different standards depends on the parameters (β and γ). For example, the JMI standard [26] is obtained with $\gamma =\beta =1/|v_{t}|$. β and γ are different in standards such as MRMR standard [28], and CIFE standard [29]. Recent studies illustrate that the higher-order feature interactions are considered to optimize feature selection standard. Therefore, we need to consider the second-order interactions between the features compared to Equation (4), such as $I(w;w_{j}|w_{i})$ [24].

(5) $$I\left(w;y_{t}\right)-\beta \sum_{w_{i}\in v_{t}}I\left(w;w_{i}\right)+\gamma \sum_{w_{i}\in v_{t}}I\left(w;w_{i}|y_{t}\right)-\delta \sum_{w_{i}\in v_{t}}\sum_{w_{j}\in v_{t};i\neq j}I\left(w;w_{j}|w_{i}\right),$$

where $\beta =\gamma =1/|v_{t}|$ and $\delta =1/|v_{t}\left|(\right|v_{t}\left|-1\right)$. Using Equation (5), the original high-dimensional MI based standard is decomposed into a group of low-dimensional MI quantities. We apply this low-dimensional approximation to the selection of lagged variables.

#### 2.2.2. Mixed Search Strategy

An applicable search strategy is important for building a mixed embedding vector. Because the greedy search strategy has high computational efficiency and good practicability, it has become the preferred strategy for embedding. However, the greedy strategy uses a sequential forward approach to select lagged variables, which rely heavily on the initial embedded vector. That is to say, the initial embedded vector is not accurate and the subsequent selection will get worse.

To solve the above problem, we present a mixed strategy to avoid inaccuracies in the initial embedding. The mixed strategy consists of two strategies: the traversal strategy and the greedy strategy. The application of the strategy is determined by defining a strategy adjustment factor *m*. Assuming that a number of iterations is *k*, the traversal strategy is applied when 1<k≤m. For example, when using the traversal strategy, it is necessary to calculate the possible combinations of all lagged variables before determining the mixed embedding vector of the current step. That is to say, we need to calculate $C_{K*L}^{k}$ combinations in total, and then select the combination of the largest conditional mutual information as the mixed embedding vector of the current step. The greedy strategy is applied when k>m. This strategy is the same as the one used by the PMIME method.

#### 2.2.3. LM-PMIME Method

**Figure 1:**
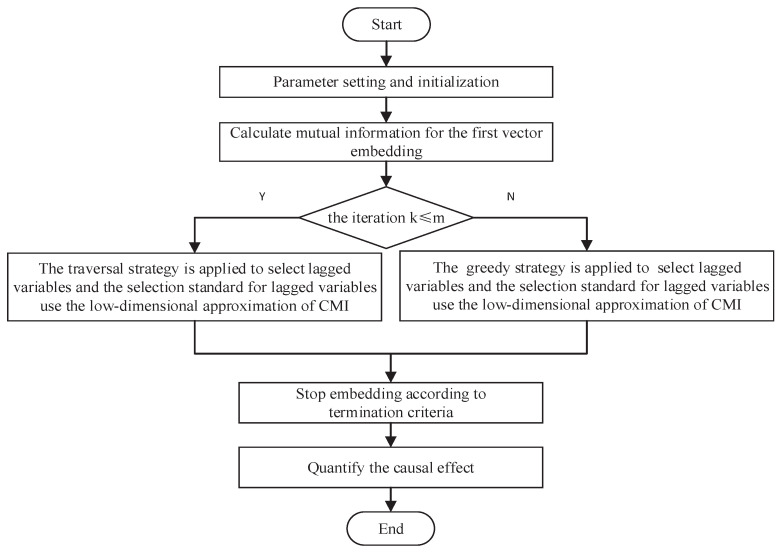
The flowchart of the low-dimensional approximation of CMI and the mixed search strategy(LM)-partial conditional mutual information from mixed embedding (PMIME) method.

We propose the LM-PMIME method to detect the directional coupling in multivariable time series according to the low-dimensional approximation of CMI and the mixed search strategy. In the LM-PMIME method, the mixed strategy determines the way to select lagged variables. Meanwhile, whether the variable will be embedded depends on the low dimensional approximation of CMI. Figure 1 illustrates the flow of the LM-PMIME method.

The detailed LM-PMIME method is as follows:

1. Initialize an empty embedding vector $v{}_{t}^{0}=\emptyset$.
2. At the first iteration *k* = 1, the embedding vector $w{}_{t}^{1}\in W_{t}$ is selected most related to $y_{t}$:
  (6) $$w{}_{t}^{1}=\underset{w\in W_{t}}{\arg\max}I\left(y_{t};w\right)$$
  Then we have $v{}_{t}^{1}=\left[w{}_{t}^{1}\right]$.
3. At the iteration 1<k≤m, $w{}_{t}^{k}$ will be selected by a standard through calculating the maximum value of the low dimensional approximation of CMI.
  (7) $$\begin{array}{c} w{}_{t}^{k}\;=\;\underset{w\in W_{t}}{\arg\max}I\left(w;y_{t}\right)\;-\;\beta \sum_{w_{i}\in v{}_{t}^{k-1}}I\left(w;w_{i}\right)\;+\;\gamma \sum_{w_{i}\in v{}_{t}^{k-1}}I\left(w;w_{i}|y_{t}\right)\;-\;\delta \sum_{w_{i}^{}\in v{}_{t}^{k-1}}\sum_{w_{j}^{}\in v{}_{t}^{k-1};i\neq j}I\left(w;w_{j}|w_{i}\right), \end{array}$$
  where $\beta =\gamma =1/|v_{t}|$ and $\delta =1/|v_{t}\left|(\right|v_{t}\left|-1\right)$. The traversal strategy is applied to select $v{}_{t}^{k}$, i.e., at the iteration k=4 and m=5, $v{}_{t}^{4}$ needs to be selected. First, clear the already embedded vector $v{}_{t}^{3}$ and calculate $C_{K*L}^{4}$ combinations in total. Then select the combination of largest conditional mutual information as $v{}_{t}^{4}$ of the current step. Finally, k=k+1.
4. At the iteration k>m, greedy strategy is used. Each $w{}_{t}^{k}$ will be embedded in the already embedded vector $v{}_{t}^{k-1}$ until the process stops. The standard of low dimensional approximation is still used before stopping.
5. The termination criterion is quantified as:
  (8) $$I\left(y_{t};v{}_{t}^{k-1}\right)/I\left(y_{t};v{}_{t}^{k}\right)>A,$$
  where the threshold A<1 and threshold *A* near 1, e.g., A=0.95, allows the inclusion of a new component in the mixed embedding vector even if the augmented vector explains very little of the information on $y_{t}$ that was not explained at the previous step. Although the statistical significance threshold α is widely used [32,33], the accuracy of causal analysis is lower than the threshold *A* and the amount of calculation is large [15,16]. Therefore, we employ the threshold *A* for non-uniform embedding. The general value of *A* is 0.95 or 0.97 in [15,16]. That is, the additional information of $w{}_{t}^{k}$ selected at the iteration *k* is not large enough. The embedding process will stop and we have the mixed embedding vector $v_{t}=v{}_{t}^{k-1}$. In addition, any combination of the lagged variables $X,Y,Z_{1},\ldots ,Z_{K-2}$ may be included in $v_{t}$.
6. To calculate the causality strength of *X* on *Y* conditioned by the other variables in *Z*, the index is defined as:
  (9) $$R{}_{X\to Y|Z}=\frac{I(y_{t};v{}_{t}^{x}|v{}_{t}^{y},v{}_{t}^{z})}{I(y_{t};v{}_{t})},$$
  where $v{}_{t}^{x}$ represents the component of *X* in $v{}_{t}$. It is the same with $v{}_{t}^{y}$ and $v{}_{t}^{z}$. The causality strength of *X* to *Y* depends on the components of *X* in $v_{t}^{x}$. The presence of components of *X* in the mixed embedding vector shows that *X* has effect on *Y* and then the derived causality strength $R_{X\to Y|Z}$ is positive, whereas the absence shows no effect and causality strength $R_{X\to Y|Z}$ is exactly zero. In addition, the $R_{X\to Y|Z}$ is considered significant if it is positive in the PMIME method and proposed method.

## 3. Simulation Study

To evaluate the effectiveness of the LM-PMIME, a series of synthetic time series from linear stochastic, nonlinear stochastic and chaotic systems are applied to compare the effectiveness of the traditional PMIME method. The effects of data length and coupling strength are considered in the numerical experiment. To show the performance of the low-dimensional approximation of CMI, the M-PMIME method only with the improvement of mixed strategy is added for comparison.

In the above models, the accuracy of the estimated mutual information is vital for embedding vector selection [28]. The two most common methods for estimating mutual information are the histogram and kernel methods. The former one is time efficient but not highly accurate [34]. The latter one has higher accuracy but comes with huge computational pressure [35]. We applied the k-nearest neighbors (k-NNs) method to estimate mutual information because the k-NNs estimator is suitable for high-dimensional data [36].

We calculate all methods on 100 realizations from each system to assess statistically the evaluation indicators (sensitivity, specificity, and F1 score). The connections between variables are classified as coupled directions and uncoupled directions to compute the confusion matrix.

### 3.1. Linear Multivariate Stochastic Process

The linear vector autoregressive (VAR) process is composed of order 4 in 5 time series (model 1 in [37]).

(10) $$\left\{\begin{array}{c} x_{1,t}=0.4x_{1,t-1}-0.5x_{1,t-2}+0.4x_{5,t-1}+e_{1,t}\\ x_{2,t}=0.4x_{2,t-1}-0.3x_{1,t-4}+0.4x_{5,t-2}+e_{2,t}\\ x_{3,t}=0.5x_{3,t-1}-0.7x_{3,t-2}-0.3x_{5,t-3}+e_{3,t}\\ x_{4,t}=0.8x_{4,t-3}+0.4x_{1,t-2}+0.3x_{2,t-2}+e_{4,t}\\ x_{5,t}=0.7x_{5,t-1}-0.5x_{5,t-2}-0.4x_{4,t-1}+e_{5,t} \end{array}\right.$$

where $e_{i,t}$, i=1,⋯,5, are Gaussian noise. $X_{1}\to X_{2}$, $X_{1}\to X_{4}$, $X_{2}\to X_{4}$, $X_{4}\to X_{5}$, $X_{5}\to X_{1}$, $X_{5}\to X_{2}$, and $X_{5}\to X_{3}$ are the true causal connections in this process.

**Figure 2:**
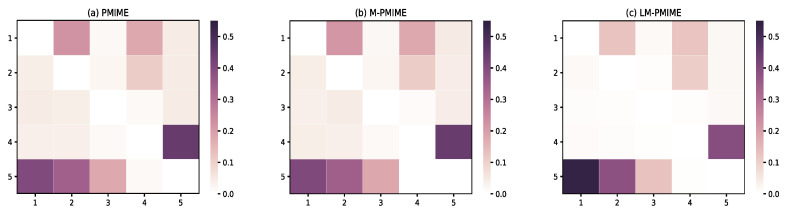
Matrix representation of causality for the linear vector autoregressive (VAR) process. Retrieved by (**a**) traditional PMIME method, (**b**) mixed search strategy (M)-PMIME method, (**c**) and LM-PMIME method with k-nearest neighbors (k-NNs) estimator. The length of the time series is 512. m=2 is used for the M-PMIME method and the LM-PMIME method. The remaining parameters of the three methods are the same (L=6,A=0.97). Color maps for the mean values of coupling measurements are obtained from 100 realizations of the linear VAR process. The direction of causal influence is from row to column in the matrix. The true causal connections in this linear VAR process are at the matrix elements (1, 2), (1, 4), (2, 4), (4, 5), (5, 1), (5, 2) and (5, 3).

We use A=0.97 and L=6, which matches the larger lag for the three methods in the process. In addition, the LM-PMIME method and the M-PMIME method use the parameter m=2. The results from the linear VAR process with the time series length of 512 are shown in Figure 2. The direction of the causal effect is from row to column in the matrix representation, e.g., the causal connection $X_{1}\to X_{2}$ is represented as (1, 2) in the matrix representation. Hence, true causal relationships in the process are (1, 2), (1, 4), (2, 4), (4, 5), (5, 1), (5, 2) and (5, 3) at the matrix elements. The mean values of coupling measured by the three methods are positive and high on these matrix elements. It is proved that the three methods have good sensitivity to true couplings. However, Figure 2 shows that there are lots of false positives in the traditional method using high-dimensional CMI. In contrast, the LM-PMIME method has better results than the other two methods, because the method reduces false positives. The sensitivity, specificity, and F1 scores are obtained from 100 realizations of linear VAR process with varying length of time series. The values of the specific indicators are listed in Table 1. The F1 score of the LM-PMIME method has better results on the linear VAR process with different time series lengths. Furthermore, the F1 score calculated by the LM-PMIME method increases as the time series length increases. These better results are likely due to the great improvement of specificity by the proposed method. At the same time, the F1 score reflects that the PMIME method and M-PMIME method have achieved similar results in the linear VAR process. It shows that the mixed strategy does not work in this process. However, the following experiments show that the mixed strategy works well on the chaotic system.

### 3.2. Nonlinear Multivariate Stochastic Process

The nonlinear VAR process is of order 1 in three variables ${\mathrm{NLVAR}}_{3}\left(1\right)$ (model 7 in [38]).

(11) $$\left\{\begin{array}{c} x_{1,t}=3.4x_{1,t-1}\left(1-x_{1,t-1}^{2}\right)e^{-x_{1,t-1}^{2}}+0.4e_{1,t}\\ x_{2,t}=3.4x_{2,t-1}\left(1-x_{2,t-1}^{2}\right)e^{-x_{2,t-1}^{2}}+0.5x_{1,t-1}x_{2,t-1}+0.4e_{2,t}\\ x_{3,t}=3.4x_{3,t-1}\left(1-x_{3,t-1}^{2}\right)e^{-x_{3,t-1}^{2}}+0.3x_{2,t-1}+0.5x_{1,t-1}^{2}+0.4e_{3,t} \end{array}\right.$$

**Figure 3:**
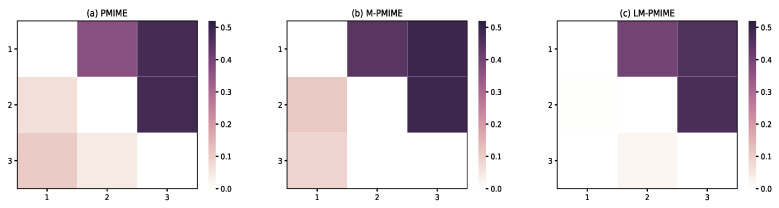
Matrix representation of causality for ${\mathrm{NLVAR}}_{3}\left(1\right)$. Retrieved by (**a**) traditional PMIME method, (**b**) M-PMIME method, (**c**) and LM-PMIME method with k-NNs estimator. The time series length is 512. m=3 is used for the M-PMIME method and the LM-PMIME method. The remaining parameters of the three methods are the same (L=6, A=0.97). Color maps for the mean values of coupling measurements are obtained from 100 realizations of ${\mathrm{NLVAR}}_{3}\left(1\right)$. The true causal connections in ${\mathrm{NLVAR}}_{3}\left(1\right)$ are at the matrix elements (1,2), (1,3), (2,3).

The true causal connections in NLVAR3 (1) are $X_{1}\to X_{2}$, $X_{1}\to X_{3}$, $X_{2}\to X_{3}$. The results obtained from 100 realizations of the nonlinear VAR process are shown in Figure 3 for n=512, A=0.97, L=6. The strategy adjustment factor m=3 determines the application of the strategies for the LM-PMIME method and M-PMIME method. The true causal connections in ${\mathrm{NLVAR}}_{3}\left(1\right)$ are represented at the matrix elements (1,2), (1,3), and (2,3). For all methods, the mean values of coupling measurements on these matrix elements are positive and high. It turns out that all methods have good sensitivity to true couplings. But there are many false positives in the traditional methods using high-dimensional CMI. Hence, the LM-PMIME method significantly outperforms the others. The evaluation indicators are obtained from ${\mathrm{NLVAR}}_{3}\left(1\right)$ by increasing the time series length. The values of the specific indicators are listed in Table 2. The F1 score of LM-PMIME method has better results on ${\mathrm{NLVAR}}_{3}\left(1\right)$ with different time series lengths. In addition, the F1 score will increase as the time series length increases. The low-dimensional approximation of CMI can greatly improve specificity, although mixed strategy does not work in ${\mathrm{NLVAR}}_{3}\left(1\right)$.

### 3.3. Coupled Henon Maps

The system of *K* coupled chaotic Henon maps (model 6 in [16]) defined as

(12) $$\left\{\begin{array}{c} x_{1,t}=1.4-x_{1,t-1}^{2}+0.3x_{1,t-2}\\ x_{i,t}=1.4-{\left(Cx_{i-1,t-1}+\left(1-C\right)x_{i,t-1}\right)}^{2}+0.3x_{i,t-2}\;for\;i=2,\cdots \;,K \end{array}\right.$$

**Figure 4:**
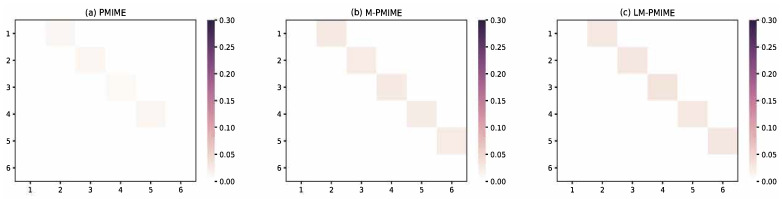
Matrix representation of causality for K=6 variables of the coupled Henon maps (C=0.1). Retrieved by (**a**) traditional PMIME method, (**b**) M-PMIME method, (**c**) and LM-PMIME method with k-NNs estimator. The time series length is 1024. m=2 is used for the M-PMIME method and the LM-PMIME method. The remaining parameters of the three methods are the same (L=5, A=0.95). Color maps for the mean values of coupling measurements are obtained from 100 realizations of the coupled Henon maps. The true causal connections in the coupled Henon maps are at the matrix elements (i−1,i), where i=2,⋯,6.

**Figure 5:**
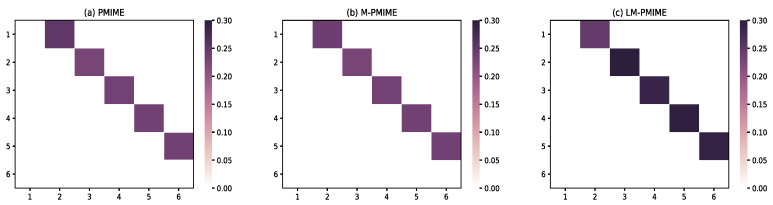
Matrix representation of causality for K=6 variables of the coupled Henon maps (C=0.3). Retrieved by (**a**) traditional PMIME method, (**b**) M-PMIME method, (**c**) and LM-PMIME method with k-NNs estimator. The time series length is 1024. m=2 is used for the M-PMIME method and the LM-PMIME method. The remaining parameters of the three methods are the same (L=5, A=0.95). Color maps for the mean values of coupling measurements are obtained from 100 realizations of the coupled Henon maps. The true causal connections in the coupled Henon maps are at the matrix elements (i−1,i), where i=2,⋯,6.

$X_{i-1}\to X_{i}$, where i=2,⋯,K, are the true causal connections in the *K* coupled chaotic Henon maps. The results from 100 realizations of the coupled Henon maps with the coupling strength C=0.1 are shown in Figure 4 for n=1024, K=6, A=0.95, L=5, m=2. In addition to this, the results of only changing the coupling strength C=0.3 are shown in Figure 5. The true causal connections in the coupled Henon maps are at the matrix elements (i−1,i), where i=2,⋯,6. There is almost no false positive for all methods. However, Figure 4 and Figure 5 illustrate that the proposed methods have better performance than the traditional method when there are true causal connections. All methods will detect stronger causal connections as the coupling strength *C* of the system increases. The evaluation indicators are obtained from coupled Henon maps with the variables *K* from 3 to 9. The values of the specific indicators are listed in Table 3 and Table 4. The results show that the F1 score of the LM-PMIME method is higher than the others when the coupling strength is low. Although the F1 score may be affected by the number of variables *K* in the simulation experiments, the F1 score for the LM-PMIME method is above 0.9. The LM-PMIME method and the M-PMIME method greatly improve the specificity, especially the former method. It is proved that both low-dimensional approximation of CMI and the mixed strategies play an important role in coupled Henon maps when the coupling strength is low.

### 3.4. Coupled Lorenz System

The chaotic system of three coupled identical Lorenz oscillators (model 5 in [16]) is defined as

(13) $$\left\{\begin{array}{c} \dot{x_{1}}=-10x_{1}+10y_{1},\;\dot{x_{i}}=-10x_{i}+10y_{i}+C\left(x_{i-1}-x_{i}\right),\\ \dot{y_{1}}=-x_{1}z_{1}+28x_{1}-y_{1},\;\dot{y_{i}}=-x_{i}z_{i}+28x_{i}-y_{i},\\ \dot{z_{1}}=x_{1}y_{1}-\frac{8}{3}z_{1},\;\dot{z_{i}}=x_{i}y_{i}-\frac{8}{3}z_{i}, \end{array}\right.$$

where i=2,3. The differential equations by the explicit Runge-Kutta (4,5) method are solved in MATLAB. The true causal connections in the three coupled Lorenz oscillators are $X_{i-1}\to X_{i}$, where i=2,3.

**Figure 6:**
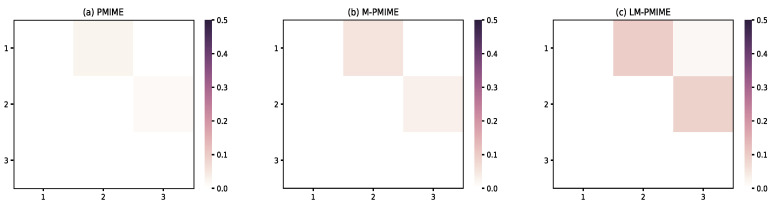
Matrix representation of causality for the three coupled Lorenz oscillators. Retrieved by (**a**) traditional PMIME method, (**b**) M-PMIME method, (**c**) and LM-PMIME method with k-NNs estimator. The length of the time series is 512 with coupling strength C=3. m=3 is used for the M-PMIME method and the LM-PMIME method. The remaining parameters of the three methods are the same (L=5, A=0.95). Color maps for the mean values of coupling measurements are obtained from 100 realizations of the three coupled Lorenz oscillators. The true causal connections in the three coupled Lorenz oscillators are at the matrix elements (i−1,i), where i=2,3.

The results from 100 realizations of the three coupled Lorenz oscillators with the coupling strength C=3 are shown in Figure 6, for n=512, A=0.95, L=5, m=3. In addition, The evaluation indicators are listed in Table 5. The values of the specific indicators are obtained from the Lorenz oscillators with varying time series length and the remaining parameters are the same. The F1 scores of the proposed methods are much higher than the traditional PMIME method. The M-PMIME method performs best when the time series is short. That is to say, the mixed strategy plays a role in improving the F1 score. However, the F1 score of the LM-PMIME method is the highest as the time series length increases.

**Figure 7:**
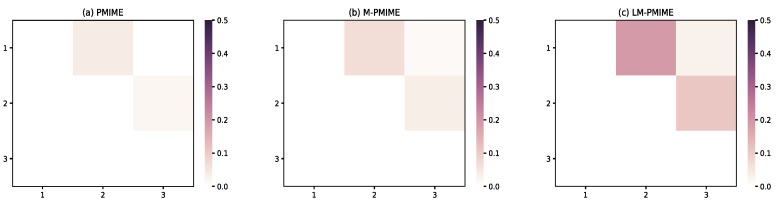
Matrix representation of causality for the three coupled Lorenz oscillators. Retrieved by (**a**) traditional PMIME method, (**b**) M-PMIME method, (**c**) and LM-PMIME method with k-NNs estimator. The length of the time series is 512 with coupling strength C=5. m=3 is used for the M-PMIME method and the LM-PMIME method. The remaining parameters of the three methods are the same (L=5, A=0.95). Color maps for the mean values of coupling measurements are obtained from 100 realizations of the three coupled Lorenz oscillators. The direction of causal influence is from row to column in the matrix. The true causal connections in the three coupled Lorenz oscillators are at the matrix elements (i−1,i), where i=2,3.

The evaluation indicators are obtained from 100 realizations of the Lorenz oscillators with coupling strength *C* from 1 to 5 for the three different methods. The time series length is 512 and A=0.95, L=5, m=3. The values of the indicators are listed in Table 6. The results show that the LM-PMIME method performs best when the *C* is low, such as *C* = 1. Although the F1 score of the traditional PMIME method increases as the coupling strength *C* increases, it is still much worse than the proposed methods. Figure 7 is the matrix representation of causality for the three coupled Lorenz oscillators with coupling strength C=5. The true causal connections are (i−1,i) in the matrix elements, where i=2,3. Only for the LM-PMIME method, the mean values of coupling measurements on these matrix elements are positive and high.

## 4. Application to Epilespy ECoG Signals

In the study, we show the applicability of the LM-PMIME method to actual electrocorticographic (ECoG) to explore key contacts of the human subject with intractable epilepsy. A public dataset from a 39-year-old woman with medically refractory complex partial seizures is used. The dataset contains 8 seizure epochs and eight pre-seizure epochs. Each epoch contains 76-time series obtained from the eight-by-eight electrode grid and two depth electrodes with six contacts each. In addition, the duration of each epoch is 10 s and the length of each time series is 4000 (see [39] for more details). The data is recorded at 400 Hz, which is downsampled to 100 Hz.

**Figure 8:**
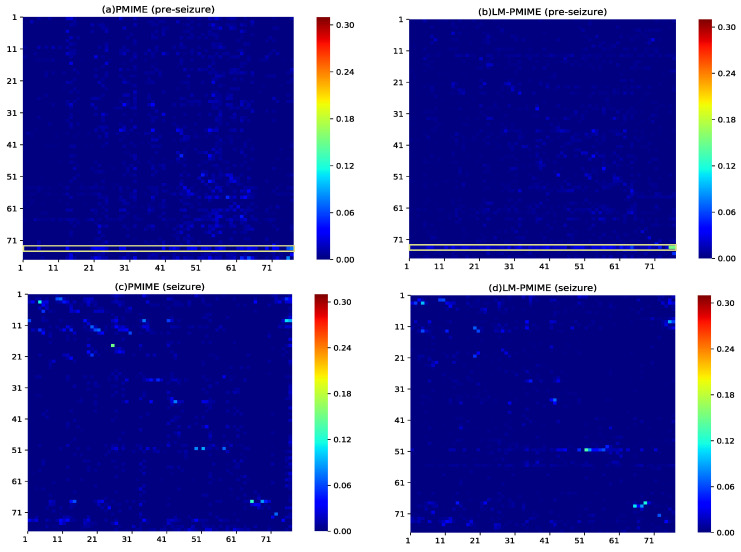
Results for multivariate electrocorticographic (ECoG) data. Matrices of causalities reflect the pre-seizure state (**top**) and the seizure state (**bottom**)) estimated by the PMIME method and the LM-PMIME method. The causal strengths are averaged (the mean values of the coupling measurements over all epochs in the same physiological state). Contacts 1 to 64 belong to an eight-by-eight electrode grid, and contacts 65 to 76 belong to two depth electrodes. The direction of causal influence is from row to column in the matrices. The brighter colors indicate more significant values. The key contact is marked by a rectangular box. The parameter A=0.95 and m=2 are set for the different methods.

**Figure 9:**
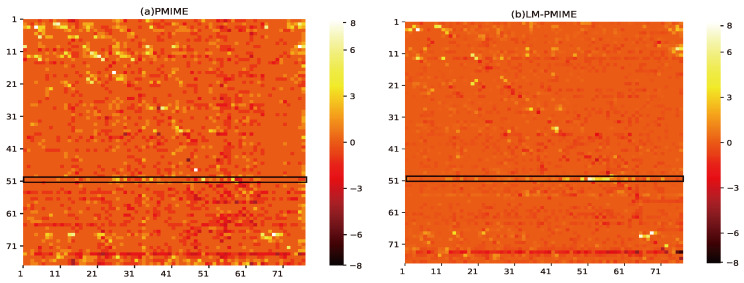
Results for multivariate ECoG data. Matrices reflect the difference of total numbers of significant connections between the seizure state and the pre-seizure state (seizure minus pre-seizure). The numbers are respectively summed from 8 seizure epochs and eight pre-seizure epochs. Contacts 1 to 64 belong to an eight-by-eight electrode grid, and contacts 65 to 76 belong to two depth electrodes. The brighter colors indicate more significant values. The key contact is marked by a rectangular box. The parameter A=0.95 and m=2 are set for the different methods.

We use PMIME method and LM-PMIME method to analyze the seizure data and the pre-seizure data, respectively. To assess the causal matrices of different physiological states estimated by each method, we compute the average causal strengths (the mean values of the coupling measurements over all epochs in the same physiological state) as shown in Figure 8. The brighter the colors are, the more signifincant causal connections are. As a result, it is obvious from the causal matrics of LM-PMIME method that contact 73 has more impact on the other contacts, highlighting that it is the key contact in the pre-seizure data (see Figure 8b). Figure 8 illustrates the difference of total numbers of significant connections between the seizure state and the pre-seizure state. The proposed method highlights the key contact 50 (see Figure 9b) and these discovered key contacts are consistent with many researchers [39,40,41].

The LM-PMIME method gives an obvious causal driver located at the contact 73 from the second depth electrode strip in the pre-seizure data shown in Figure 8. Therefore, the contact 73 may be associated with seizures. Although not yet clinically observable, it has been suggested that the second depth electrode primarily affects cortical activity in [40,41]. In addition, the proposed method successfully identifies a key contact from the data: contact 50, which exhibits the most significant change in the betweenness centrality. The contact is considered the primary target of therapeutic intervention in [38], because contacts with statistically significant increases in betweenness centrality may lead to seizures.

## 5. Discussion and Conclusions

In this study, we show effective modifications for the well-known non-uniform embedding method: PMIME, which quantifies causality by means of information theoretic measures. The advantage of the non-uniform embedding compared with uniform embedding is that it can reduce the dimension of the state space by selecting the relevant variables which contribute the most to explain the target variable. Therefore, it has been proved that the non-uniform embedding process is more flexible for state-space reconstruction [11,15,42]. However, there are still some obstacles to estimating high-dimensional CMI and constructing an optimal mixed embedding vector in the traditional non-uniform embedding methods. The proposed LM-PMIME method overcomes the above shortcomings of traditional methods. The major contribution of the proposed LM-PMIME method, which is based on the low-dimensional approximation of conditional mutual information and the mixed search strategy, is that improves the traditional non-uniform embedding methods. The curse of dimensionality is avoided by replacing the original estimate with a low-dimensional approximation of CMI. In addition, a mixed strategy instead of the greedy strategy is used as an embedded strategy to solve the problem of initial embedding inaccuracy. Hence, the mixed embedding vector becomes more parsimonious by maximizing the correlation with the target variable and minimizing the redundancy between the selected variables. In order to form the optimal mixed embedded vector, there are also other propositions. For example, in [22] a preselection scheme for subsets of causal predictors is used to search an optimal subset and detect the synergetic variables. In addition, many researchers adopt the OCE algorithm [43] or the PCMCI [30] algorithm to estimate the causal graphs. Different from these preselection methods, the LM-PMIME method relies on both the low-dimensional approximation and the mixed search strategy to improve the conditions. In all simulation systems, the LM-PMIME method performs better than the traditional methods according to the F1 score. Because of the complexity of chaotic systems, true causality is often difficult to detect. However, the LM-PMIME method significantly improves the sensitivity in chaotic systems. In the remaining simulation systems, the LM-PMIME method reduces false positives and increases the specificity. The experiments also adopt the comparison method M-PMIME, which improves the search strategy without using low-dimensional approximation. By the M-PMIME method, it can be found that the mixed search strategy works well in chaotic systems, especially the systems with low coupling strengths. In addition, the low-dimensional approximation of CMI plays an important role in linear and nonlinear systems. Therefore, we combine both the low-dimensional approximation of CMI and the mixed search strategy to form a new non-uniform embedding method LM-PMIME for multivariate time series. Although the LM-PMIME method works better than the traditional method, there are limitations to the proposed method. It still does not exceed the efficiency of the standard uniform embedding methods under the non-uniform embedding framework. In addition, the strategy adjustment factor *m* is larger, the traversal strategy is used more times in iterations. The main role of the traversal strategy is to correct the initial embedding inaccuracy, which is often time-consuming. Therefore, the recommended value is m<5 and the simulation results generally take m=2 or m=3 to achieve better results. This is because a smaller value of *m* can reduce the computation time and it is enough to optimize the initial embedding.

Generally speaking, the proposed low dimensional approximation of CMI and mixed search strategy improve the non-uniform embedding process, which is also applicable to other causal analysis methods based on non-uniform embedding [32]. In this study, we present the LM-PMIME method to detect directional coupling for multivariate time series. The effectiveness and applicability of the LM-PMIME method are demonstrated by a large number of experiments. The LM-PMIME method works well for multivariate time series with weak coupling strengths. In addition, the proposed LM-PMIME method, a causal analysis method, has great potential to be adopted in other applications, e.g., prediction of dynamic systems. We will further study the non-uniform embedding method and extend its applications.

## Figures and Tables

**Table 1:** Evaluation indicators are obtained from 100 realizations of linear VAR process with varying time series length for the three different methods. A=0.97 and L=6 are the parameters common to the three methods. In addition, the LM-PMIME method and the M-PMIME method use the parameter m=2.

	Sensitivity	Specificity	F1 Score
		n=256	
PMIME	0.988	0.492	0.600
M-PMIME	0.989	0.481	0.596
LM-PMIME	0.797	0.741	0.647
		n=512	
PMIME	1.000	0.567	0.643
M-PMIME	0.994	0.727	0.645
LM-PMIME	0.855	0.763	0.693
		n=1024	
PMIME	1.000	0.697	0.719
M-PMIME	0.940	0.729	0.713
LM-PMIME	0.877	0.807	0.739

**Table 2:** Evaluation indicators are obtained from 100 realizations of ${\mathrm{NLVAR}}_{3}\left(1\right)$ with varying time series length for the three different methods. A=0.97 and L=6 are the parameters common to the three methods. In addition, the LM-PMIME method and the M-PMIME method use the parameter m=3.

	Sensitivity	Specificity	F1 Score
		n=256	
PMIME	0.973	0.650	0.737
M-PMIME	0.976	0.615	0.712
LM-PMIME	0.860	0.844	0.792
		n=512	
PMIME	1.000	0.681	0.758
M-PMIME	1.000	0.662	0.748
LM-PMIME	0.950	0.887	0.873
		n=1024	
PMIME	1.000	0.860	0.877
M-PMIME	1.000	0.790	0.827
LM-PMIME	0.989	0.892	0.896

**Table 3:** Evaluation indicators are obtained from 100 realizations of *K* variables of the Henon maps (C=0.1) for the three different methods. A=0.95 and L=5 are the parameters common to the three methods. In addition, the LM-PMIME method and the M-PMIME method use the parameter m=2.

	Sensitivity	Specificity	F1 Score
		K=3	
PMIME	0.175	1.000	0.297
M-PMIME	0.715	1.000	0.834
LM-PMIME	0.945	1.000	0.972
		K=6	
PMIME	0.217	1.000	0.357
M-PMIME	0.674	1.000	0.806
LM-PMIME	0.926	0.998	0.950
		K=9	
PMIME	0.204	1.000	0.338
M-PMIME	0.700	1.000	0.824
LM-PMIME	0.895	0.998	0.904

**Table 4:** Evaluation indicators are obtained from 100 realizations of *K* variables of the Henon maps (C=0.3) for the three different methods. A=0.95 and L=5 are the parameters common to the three methods. In addition, the LM-PMIME method and the M-PMIME method use the parameter m=2.

	Sensitivity	Specificity	F1 Score
		K=3	
PMIME	1.000	1.000	1.000
M-PMIME	1.000	1.000	1.000
LM-PMIME	1.000	1.000	1.000
		K=6	
PMIME	1.000	1.000	1.000
M-PMIME	1.000	1.000	1.000
LM-PMIME	1.000	1.000	1.000
		K=9	
PMIME	1.000	1.000	1.000
M-PMIME	1.000	1.000	1.000
LM-PMIME	1.000	1.000	1.000

**Table 5:** Evaluation indicators are obtained from 100 realizations of the three coupled Lorenz oscillators (C=3) with varying time series length for the three different methods. A=0.95 and L=5 are the parameters common to the three methods. In addition, the LM-PMIME method and the M-PMIME method use the parameter m=3.

	Sensitivity	Specificity	F1 Score
		n=256	
PMIME	0.225	0.997	0.364
M-PMIME	0.660	0.863	0.617
LM-PMIME	0.805	0.665	0.541
		n=512	
PMIME	0.185	1.000	0.312
M-PMIME	0.640	0.913	0.658
LM-PMIME	0.875	0.743	0.631
		n=1024	
PMIME	0.175	1.000	0.297
M-PMIME	0.670	0.909	0.674
LM-PMIME	0.970	0.756	0.687

**Table 6:** Evaluation indicators are obtained from 100 realizations of the three coupled Lorenz oscillators (n=512) with coupling strength *C* from 1 to 5 for the three different methods. A=0.95 and L=5 are the parameters common to the three methods. In addition, the LM-PMIME method and the M-PMIME method use the parameter m=3.

	Sensitivity	Specificity	F1 Score
		C=1	
PMIME	0.000	1.000	0.000
M-PMIME	0.155	0.926	0.221
LM-PMIME	0.375	0.830	0.381
		C=2	
PMIME	0.075	1.000	0.141
M-PMIME	0.565	0.893	0.583
LM-PMIME	0.825	0.765	0.623
		C=3	
PMIME	0.185	1.000	0.312
M-PMIME	0.640	0.913	0.658
LM-PMIME	0.875	0.743	0.631
		C=4	
PMIME	0.260	1.000	0.413
M-PMIME	0.740	0.892	0.698
LM-PMIME	0.920	0.710	0.627
		C=5	
PMIME	0.320	0.997	0.481
M-PMIME	0.725	0.873	0.660
LM-PMIME	0.960	0.731	0.661
